# Supplementary material for: Long-term visual prognosis and characteristics of recurrent retinal detachment after silicone oil removal
Source: PLoS One. 2023 Feb 8;18(2):e0265162. doi: 10.1371/journal.pone.0265162 (PMC9907833; doi:10.1371/journal.pone.0265162)
Supplement: Attachment — Submitted filename: Response to reviewers.docx [file pone.0265162.s002.docx]

**Response to reviewers**

Review Comments to the Author

Please use the space provided to explain your answers to the questions above. You may also include additional comments for the author, including concerns about dual publication, research ethics, or publication ethics. (Please upload your review as an attachment if it exceeds 20,000 characters)

Reviewer #1: The authors present a large cohort of RD eyes treated with PPV with silicon oil tamponade.
They report 8.9% re-detachment rate after silicon oil removal in a 50 month follow up and found that this was associated with worse final BCVA compared to the non re-detached group.
Longer period of silicon oil in the eye was associated with worse visual outcome potentially due to higher corneal opacity rates. The authors are advised to consider the following recommendations to eventually strengthen their work:

1. Lines 120-121: ‘The presence of scleral buckling or the number of mean previous retinal surgeries were not different between the primary retinal attachment group and the re-detachment group after removal of SO.’
How would the authors explain their finding that scleral buckle was not associated with lower re-detachment rates following silicon oil removal ? Lines 178-179 do not clearly and adequately elaborate on this.

In our study, the difference in the recurrence rate cannot be explained simply by the presence or absence of a buckle. However, if the PVR was removed well as planned, it would have produced good results without recurrence even if the buckle was not accompanied by sufficiently removing the inflammatory cells. Follow-up studies on matched control will be helpful.

2. Line 87: Please define ‘’exploratory data analysis’’. What statistical test was used for comparisons between 3 groups ? (<3months,3-6months, >6months)

Thanks for the details. To compare the three groups, ANOVA test and Kruskal-Wallis test were performed on continuous variables, and Chi-squared test and Fisher's exact test were performed on categorical variables.

3. Lines 80-81: Was Swept source Optical Coherence Tomography (OCT) performed in all patients since 2009?

Thanks for the sharp point. SS-OCT was introduced to the institution in 2013. SD-OCT and SS-OCT are used together in this study.

4. Line 82: Please define ‘’and the other unwanted complications development’’
Line 86: Please define stratification of macula status

Line 82: Thanks. Noted that unwanted complications include secondary glaucoma, corneal opacity, and pththisis bulbi.

Line 86: It was to confirm whether retina was re-detached through OCT, but the meaning was expressed vaguely. We deleted the ambiguous part.

5. Line 96: Please add a percentage for TRD as with RRD
The percentage for TRD is also presented. Thank you.

6. Lines 157-159: Please proof read for the proper use of English. Also, language errors could be found throughout the manuscript. Please proof read the entire work.

Thanks for the point. We have corrected the parts you mentioned and other vaguely expressed sentences.

7. Please change ‘primary attachment group’ to ‘no re-detachment group’

Thanks. As you mentioned, ’primary attachment group’ was modified to ‘no re-detachment group’.

8. The authors need to explain their finding that the presence of PVR did not increase the chance of re-detachment in this study. Lines 164-168 do not clearly and adequately elaborate on this.

Modified and supplemented. Thanks for the point.

9. Line 167: please define ‘and so on’

Thank you for pointing out the point. In addition to the contents expressed, whether the band has been raised well and if the SRF has been sufficiently removed would have influenced the results.

10. Limitations should be at the end of the discussion - not in the middle

Thanks for the details. Paragraphs related to limitations were sent to the back.

11. Abstract: ‘side effects’ should read ‘adverse events’
Abstracts occlusion ‘for too long’ is too vague. Please be more specific in your conclusions.

Thanks for the sharp point. Modified to ‘adverse events’. Ambiguous expressions have also been corrected.

12. Line 205 ‘The main purpose was to evaluate the risk factors leading to, and to examine the factors indicating, retinal re-detachment.’’ what are the conclusions of the authors ? How do they reply to their main research question ‘what are the risk factors for retinal re-detachment following SO removal?’ It seems there is no clear risk factor per the authors results. Please provide a robust conclusion in the manuscript.

Thank you. Other factors did not show any significant difference, but it was confirmed that the duration of SO tamponade had a difference in visual acuity after surgery and did not significantly affect retinal detachment. If it is determined that the retina is sufficiently stable, it is not necessary to delay the timing of SO removal.

Reviewer #2: This study adds to the literature significant information reporting on the long term visual prognosis and recurrence factors after pars plans vitrectomy and use of silicon oil for the treatment of retinal detachment. The authors in general provided enough data to support their conclusions. Furthermore, the statistical analysis seems to be well conducted. However, I would like a statistician to look at it. The following are a few points that need to be reviewed.

Major points
As far as the language is concerned, a few sentences lack consistency, clarity and syntax (e.g. lines 85-86, 95-97, 143-145, discussion), making it difficult to follow. It is advisable that the authors proofread the text to improve the flow and the readability of the manuscript. If need be, the authors could work with an expert.

The authors should rewrite the introduction of their study and give a more general scope at it. Rhegmatogenous retinal detachment is only one specific type of retinal detachment. Moreover, the other types of retinal detachment included in the study are not mentioned at all, giving the opinion that only RRD eyes participated.

Thanks for the sharp point. As pointed out, we modified the introduction section to include other types of RD.

Since a great percentage of the included patients has had at least one retinal surgery in the past, it is important to be clarified in the methods whether the RD was primarily occurred to the eyes studied or there were patients with recurrent RD that were included in the study. Moreover, it might be helpful, if the authors referred to any exclusion criteria they used, concerning the recurrent RDs (e.g. treated in other center and presented in recurrence, other primary treatment option, etc)

Thanks for your attention to detail. We mentioned in the methods that the surgery also included cases that had recurred after previous surgery. No matter where the previous surgery was done, all cases of RD were included in the study, so exclusion criteria were not specifically addressed for recurrence.

The authors should rewrite the discussion aiming at a more comprehensive presentation of previous data. They should revise and clarify a few sentences (e.g. lines 161, 163-166, 173-175, 177-179, 182, 185-186, 227) and provide previous evidence exactly according to references. They should also include references for every percentage or result they report, in order to avoid confusion and render it easier for the reader to relate their findings to previous studies. It is advisable that they firstly report the outcomes of their study and secondly refer and relate to previous findings, in order to avoid possible confusion of the presented outcomes and a “back and forth” analysis.

Thank you for the clear and accurate point. I have refined and corrected the parts you said.

An additional possible limitation of the study could be the fact that there was statistically significant difference in the three groups of different SO tamponade period, as far as BCVA before SO injection is concerned (table 4). The authors should consider how this difference could influence the last F/U findings and report it in their study (lines 221-223).

Thanks for the good point. At first glance, it can be thought that the worse the preoperative vision was, the worse the preoperative retinal condition was anatomically or functionally. It is difficult to give a clear explanation of how the surgery before SO injection influenced the final difference in visual acuity because there is a limitation of a table that compares groups by dividing the group only by tamponade period without considering other variables thoroughly. Matching all of those variables will lead to more accurate conclusions in subsequent studies.

In the last paragraph of the manuscript, the authors refer to data that are not available in the study. The authors should make the whole data they used fully available for the reader, especially when they want to emphasize on a result arose from them.

The sentence was corrected using only the figures presented. Thanks.

Minor points
Line 78: It should be clarified whether the BCVA was obtained at least six months after the vitrectomy or the SO removal (line 32).

Thanks. Corrected the sentence more clearly.

Table 2: It is depicted that a total of 370 eyes underwent concomitant scleral bucking surgery, whereas in Table 1 and in text it is reported that a total of 369 eyes underwent this procedure concomitantly.

There seems to be an error in the count. Has been modified. The concomitant buckling was done at 369eyes.

Lines 212-213: Τhe groups of this specific analysis should also be mentioned.

Thanks for the detailed point. The text has been revised to clarify the meaning.
