# Supplementary material for: Long-term visual prognosis and characteristics of recurrent retinal detachment after silicone oil removal
Source: PLoS One. 2023 Feb 8;18(2):e0265162. doi: 10.1371/journal.pone.0265162 (PMC9907833; doi:10.1371/journal.pone.0265162)
Supplement: Attachment — Submitted filename: Response to Reviewers(1).docx [file pone.0265162.s003.docx]

**Response to Reviewers**

**Reviewer #1**: The abstract should be able to stand alone. That means the factors mentioned in methods, should be also mentioned in results and thats where the conclusions should derive from. These sections should match.

In the abstract's methods the authors mention ‘Best-corrected visual acuity was obtained preoperatively and at least 6 months after the SO removal.’ yet pre-op and 6 months post-op BCVA is nowhere in the abstract’s results section.

Conversely, subgroups are mentioned in the results section that were never defined in the methods section. BCVAs need to be added in the abstract at least in parentheses. (for example after the phrase ‘The longest tamponade group showed the worst final vision after SO removal’). The ‘presence of accompanying buckles’ ‘ the number of operations’ and ‘systemic diseases’ are mentioned in methods as factors to be investigated yet are nowhere in the abstract’s results section.

Abstract conclusions: ‘not for too long’ is vague. What are the key conclusions/take home messages for the reader ?

Thans for the advice. As you pointed, the abstract was summarized and refined, and the conclusion part was revised more clearly.

The structure of the main manuscript’s discussion is still confusing. As reviewer #2 pointed out in the previous round of review ‘It is advisable that they firstly report the outcomes of their study and secondly refer and relate to previous findings, in order to avoid possible confusion of the presented outcomes and a “back and forth” analysis.’ This does not seem to have changed, the reader is struggling to identify the results of this study in a compact manner within this discussion and the flow is not easy to follow. Please revise accordingly.

Thanks for the point. The structure of the discussion section has been modified to make it easier to understand.

Minor points

Lines 82-83 should read: Either Swept source Optical Coherence Tomography (OCT) (Topcon, Japan) or spectral-domain OCT (Heidelberg, Germany), since I assume the study eyes did not get both tests and as mentioned by the authors in their point-by-point response SS-OCT was introduced in their clinic in 2013.

Modified and supplemented. Thanks for the point.

Line 113, 286: Please change ‘primary attachment group’ to ‘no re-detachment group’

Thanks for the details. It is corrected.

**Reviewer #2**: Although the revised version has rendered the manuscript of a higher quality, a few of my previous comments were not fully addressed. It is advisable that you take into consideration the following points and revise the manuscript accordingly.

1) Concerning the language of the text, a few sentences continue to lack clarity and syntax (e.g. lines in the revised paper 89-90, 99-101, 147-150). A careful proofreading of the whole text is suggested to improve the language and the flow of the manuscript. Please take into consideration the specific lines mentioned.

Thanks for the point. We have corrected the parts you mentioned and other vaguely expressed sentences.

2) Lines 50-52: References should be included. What about the exudative type of RD?

Thanks. We inserted a reference. The exudative type was not included as an indication for surgery.

3) Line 81: "Best-corrected visual acuity (BCVA) was obtained preoperatively and at least 6 months after the vitrectomy.” still contradicts line 32 in abstract “was obtained preoperatively and at least 6 months after the SO removal.” It should be clarified when the BCVA was exactly obtained.

The sentence was corrected to say that BCVA was measured once before surgery and again after 6 months of operation. Thank you.

4) Table 2: It still depicts that a total of 370 eyes underwent concomitant scleral bucking surgery, whereas, as authors report, a total of 369 eyes underwent this procedure concomitantly.

Thanks for the point. We have corrected an error.

5) Line 148: The word “between" had better be replaced with “compared to”.

Thanks for the details. Correction was made.

6)Line 171: Since an outcome of the current study is reported, it is not clear why the reference [6] is included here.

Thanks for pointing out. Removed unnecessary reference mark.

7) Lines 251-255: These results have been discussed again in the previous paragraph.

Thanks for the sharp point. Removed duplicate content.
