# Supplementary material for: Long-term visual prognosis and characteristics of recurrent retinal detachment after silicone oil removal
Source: PLoS One. 2023 Feb 8;18(2):e0265162. doi: 10.1371/journal.pone.0265162 (PMC9907833; doi:10.1371/journal.pone.0265162)
Supplement: Attachment — Submitted filename: RevierwComment(22).docx [file pone.0265162.s005.docx]

**Response to Reviewers**

**Reviewer #1**:

Abstract: The phrase ''if the retina is structurally stable enough, it is advisable to consider SO removal'' doe not derive from the authors results. What is '' structurally stable enough'' and how was it evaluated ?

Thanks for the advice. Ambiguous sentences have been corrected to convey the meaning more clearly.

Exclusion criteria: on line 78 need to be more detailed. All pre-existing diseases affecting the macula or optic nerve were excluded ? How about trace ERM ?

It has been explained in more detail.

Methods line 84: ‘’Best-corrected visual acuity (BCVA) was measured before surgery and 6 months after the vitrectomy.’’ Was BCVA not evaluated in the cases that the silicon oil removal happened more than 6 months after vitrectomy ? Was BCVA not evaluated in the last follow up visit that you say it was a mean of 56 months ? Please be consistent. Methods should say we will evaluate A, B , C, D and results should MATCH those A, B, C, D. Describe in the methods all the comparisons that you intend to present in your results

Thanks for the point. Ambiguous expressions have been corrected.

Conclusion is too general. What are the take home messages to the reader? what were the key findings in this study ? When should we remove SO and which factors show high risk of re-detachment? Answer the questions raised by your title.

The conclusion is presented more clearly. Thank you very much for your review.

**Reviewer #2:**

My previous comments were not fully addressed. The authors should take into consideration the following points.

Abstract:

Comparisons between no re-detachment and detachment groups were also conducted by the authors, though not referred in the abstract, neither in the methods nor the results sections. The authors should provide an abstract that reflects the whole methodology of their study and the investigations they conducted. They should revise the afore mentioned sections accordingly.

Thanks for the point. The descriptions were also added to the corresponding sessions.

Line 39: “The average BCVA was 1.60 ± 0.75 before SO removal and 1.29 ± 0.96 after the removal.”: it should be mentioned that these results refer to LogMAR.

Thanks for the advice. The ‘logMAR’ term was added.

In addition, preoperative BCVA measurements, i.e. before SO injection, are still not provided in results section of the abstract, as reviewer #1 pointed out in the previous round of review. They should be added.

Preoperative BCVA measurement data was added.

Moreover, as far as the abstract is concerned, P values of statistical significance should also be provided.

Patients and Methods

The authors responded to my previous comment regarding the exudative type of RD that “The exudative type was not included as an indication for surgery.” On the contrary, in methods section (lines 76-77) they report that RD cases such as RRD, tractional retinal detachment (TRD) and proliferative vitreoretinopathy (PVR) were included. In addition to that, Table 1 does not include all indications for surgery, since the sum of the percentages do not equal 100%. Were there other indications and which? The authors should clarify whether exudative type of RD was also investigated and revise the provided table with demographics in order to present complete data.

Thanks for the advice. During data analysis, traumatic RD was separately classified, and while organizing the data in the table, this part was omitted. Traumatic RD patients were re-examined, and it was confirmed that all the patients had tear or dialysis. So all of them were reclassified into the RRD group.

As I also mentioned in both previous round of reviews, it still remains unclear and confusing, when the BCVA was exactly obtained. More specifically, “after the SO removal” (as it is referred in abstract, results and discussion) means that BCVA was obtained, after the SO tamponade period had been completed, which differs among the studied eyes. In contrast, in line 84, it is mentioned that “Best-corrected visual acuity (BCVA) was measured ….6 months after the vitrectomy.”, which implies that BCVA was measured 6 months after the vitrectomy, independently of the SO tamponade period and SO removal. These two constitute two different time points. The authors should clarify when the BCVA was exactly obtained. It is advisable that abstract, methods, results and provided tables match regarding this information. Additionally, in line 116 the authors refer to the same time point using the term: “measured at the latest visit”. They had also better refer to a specific time point with the same term each time in order to avoid any misunderstanding.

Thanks for the sharp point. BCVA was measured before SO inj surgery, 1 month, before SO removal, 1, 3, 6 months, and 1 year, and also at the last visit. The visual acuity at the last visit used for data analysis was the last visual acuity tested annually after surgery.

Discussion

Line 172: “Patients with PVR and RD had relapses in 14.8% after SO removal”. This percentage is not correct. The percentage of patients with RD and present PVR (n=158) that had re-detachment after SO removal (n=13) is 8% (13/158=0.08), which differs a lot from the provided percentage, i.e. 14.8%. The authors should revise accordingly.

There seems to be some confusion when analyzing the data. Thanks for pointing out.

Lines 193-194: “which did not show a statistically significant difference in the RD recurrence rate between the group with and without the buckle band.”: the authors should modify how they present the results of their study, so that the latter absolutely reflect the precise analyses they conducted. Thus, the previous sentence had better be replaced with: “the percentage of concomitant buckling did not differ significantly between the “no re-detachment” and “re-detachment” groups.” Furthermore, this comparison concerns all types of RD and not only RD with PVR, as the referenced findings of Jost et al. The authors should revise accordingly.

Thank you. The contents of this study have been modified to be delivered more smoothly.

Lines 228-243: This paragraph remains confusing. The authors refer to results of a lot different analyses, i.e. at first they refer to the comparison between the group with reduced or stationary vision and the group with improved vision after SO removal, then to the analysis for each period of SO tamponade concerning visual acuity, corneal opacity and RD recurrence and except for that they also mention a possible limitation of their study, as far as BCVA is concerned. They should rewrite the paragraph and present the outcomes in a more accurate, sound and appropriate manner. They should also consider referring to these outcomes at the beginning of discussion section and not at the end, as pointed out in previous rounds of review, too.

We have edited the paragraph to be more accurate and appropriate. Thank you.

Lines 221-225 and lines 230-232 report the same results. My previous comment regarding these lines was not addressed. Please revise them.

The discussion section has been corrected to be more appropriate. Thank you.

Although significantly different BCVAs before SO injection were referred in discussion section, they were not presented as a possible limitation of the present study. The authors should make a comment in this section.

Thank you. The points you mentioned were specified as the limitations of this study.

Minor points

Since there are a lot of grammatical and language mistakes in the manuscript, a careful proofreading of the whole text is still needed in order to render it sound and clear. Some of my following points address precisely lines that should be revised.

Lines 31-32: “Best-corrected visual acuity (BCVA) was before surgery and 6 months after the SO removal.”: the word “obtained” should be added.

Thanks for the advice. As you pointed, the sentence was corrected.

Lines 93-94: The Mann–Whitney U test was used to compare the predefined outcome measures the SO-treated eyes.: this sentence lacks syntax and clarity. Please revise it.

The sentence was revised.

Line 102: “was the second most cause of surgery”: the word “frequent” should be added.

The word was added.

Lines 112, 115, 129-130: “primary retinal attachment group” should be replaced with “no re-detachment group”.

The sentences were corrected.

Line 188: the word “was” should be changed to “is”.

The sentence was corrected.

Line 206: it should be changed to “in contrast to”.

The sentence was revised.

Lines 209-213: “Probably in most areas of clinical surgical field today, the same equipment, instruments and similar surgical techniques were used, and the surgical experience and results of five surgeons in this study were very similar. In a further study, we also could find out the difference from a surgeon factor meanwhile environmental condition should be maintained the same.”: it is advisable that the authors revise these sentences as far as the syntax is concerned, in order to render clear enough what they imply.

Line 219: “But as our knowledge,..”: this phrase should be replaced.

The sentence has been corrected.

Line 224: “but showed a significant difference”: the sentence lacks subject.

The sentence was corrected.

Line 225: “were not arose” should be changed to “did not arise”.

The sentence was revised.

Line 225-226: “especially much higher in long term follow up group.”: the phrase should be revised.

The sentence has been corrected.

Line 235: “but since there is no adjustment for variables other than the tamponade period. It cannot be concluded.”: this should be one sentence.

The correction has been made.

Lines 263: Replace “suggest” with suggests

Thanks for the advice. The word was corrected.

Reviewer #1: Please mention specific page and lines that the changes are made, as well as the phrase that was added or changed in quotation marks for each one of the reviewers comments. Otherwise the reviewers cannot track the changes that you have or haven't made. Please avoid general phrases such as ‘’Ambiguous expressions have been corrected.’’ Be specific in your point-by-point response. Additional comments:

Abstract Methods: the phrase: After SO removal, the anatomic outcomes were compared between the re-detached group and the no re-detached group.’ needs correction. The groups mentioned were not previously defined. Anatomical outcomes between re-detached and not re-detached retinas ? These are be definition different.

Page 2, line 33

The sentence was corrected to ‘The anatomical results were compared between the group in which the retina was detached again after SO removal and the group in which the retina was not detached.’.

Abstract Methods: Subgroup analysis looking for what outcome ?

Page 2, line 36

It was indicated that subgroup analysis was performed to determine whether the duration of SO tamponade had an effect on RD recurrence.

Abstract Methods: other complications is too general. Either be specific or omit from the abstract.

Page 2, line 38

‘other complications’ were deleted.

Abstract Results: ‘’Overall, visual acuity improved after removal of oil than before surgery.’’ P-value is missing. Was this statistically significant ?

Page 2, line 44

That sentence has been deleted.

Abstract Results: ‘’There was no significant difference’’ and you show a p value of <0.001 ?? Clarify your sentence if you mean after So removal VA significantly different between the 2 groups.

Page 2, line 46

Corrected the sentence with ‘but visual acuity of re-detachment group was worse than no re-detachment group after SO removal (p<0.001)’.

Abstract Results: ‘’than the other two groups with shorter tamponade periods’’ what are the 2 other groups ??

Page 3, line 49

They are the groups with SO tamponade duration of less than 3 months and between 3 and 6 months.

Line 99: what are the 3 groups ? This has not been defined.

Page 5, line 102

They are the groups divided by the duration of the SO tamponade. Description was added.

Please write a conclusion that answers the research questions that your study intended to answer based on your results.

Reviewer #2: There are a few points that still need to be revised.

Abstract - Lines 45-47: P values of statistical significance should be provided here, too.

Page 3, line 51

P values were added.

Lines 88-89: “BCVA was measured before SO injection surgery and 1 month after the surgery, before SO removal surgery, 1, 3, 6 months, and 1 year after removal.”: The authors should not use commas if it is not necessary, because it can lead to misconceptions. Do “1, 3, 6 months” refer to the period before or after SO removal? In the period before SO removal, was BCVA evaluated only one month after SO injection surgery or at additional time points, e.g. 1, 3 and 6 months? These points remain confusing and need to be clarified.

If the evaluation of BCVA before SO removal was only conducted one month after the SO injection surgery, independently from the SO tamponade period of each group, it should be mentioned that whenever “Before SO removal” is used in the provided tables and results, it refers to that specific time point, i.e. one month after SO injection surgery. It is advisable that the authors mention this correspondence.

Page 4, line 93

BCVA was measured when hospitalized for SO removal surgery, and was measured at 1 month, 3 months, 6 months, and 1 year after removal, respectively. The manuscript was revised to reflect these contents.

Lines 88-90: Since the time point of last follow up differed among patients, it should be included as a possible limitation of the present study. BCVA at last follow up would have been more appropriately comparable between groups, if the last follow up referred to a specific time point for all patients, e.g. 6 months after SO removal. Other factors could possibly have affected BCVA within longer follow up periods.

Page 13, line 253

The points you figured out were additionally described as limitations of the study. Thank you.

Line 98: “measures" should be replaced with measurements.

Page 5, line 104

Thanks for the comment. As you said, I modified it with measurements.

Lines 215-217: As I have already commented, this comparison of the study concerns all types of RD and not only RD with PVR. This difference should be mentioned and rendered clear enough to the reader, since all other studies, that are referenced at this paragraph, investigated RD with PVR cases. The authors could first refer to their outcomes (lines 215-217) and then present the results of other studies, that were focused precisely on RD with PVR.

Furthermore, Deaner et al and Garweg et al reported specifically outcomes about eyes with retinal detachment complicated by proliferative vitreoretinopathy, a fact that should also be reported.

Lines 243-244: It should be replaced with: “An additional limitation of this study is the fact that visual acuity before SO injection was significantly different in the three groups of different SO tamponade period and this could have affected the visual prognosis after surgery.”

Page 13, line 250

Edited as you mentioned. thank you.
